# Joint Effects of Asymmetric Payoff and Reciprocity Mechanisms on Collective Cooperation in Water Sharing Interactions: A Game Theoretic Perspective

**DOI:** 10.1371/journal.pone.0073793

**Published:** 2013-08-28

**Authors:** Cho Nam Ng, Raymond Yu Wang, Tianjie Zhao

**Affiliations:** 1 Department of Geography, The University of Hong Kong, Pokfulam, Hong Kong; 2 Institute of Remote Sensing and Digital Earth, Chinese Academy of Sciences, Beijing, China; Universidad Carlos III de Madrid, Spain

## Abstract

Common-pool resource (CPR) dilemmas distinguish themselves from general public good problems by encompassing both social and physical features. This paper examines how a physical mechanism, namely asymmetric payoff; and a social mechanism, reciprocity; simultaneously affect collective cooperation in theoretical water sharing interactions. We present an iterative N-person game theoretic model to investigate the joint effects of these two mechanisms in a linear fully connected river system under three information assumptions. From a simple evolutionary perspective, this paper quantitatively addresses the conditions for Nash Equilibrium in which collective cooperation might be established. The results suggest that direct reciprocity increases every actor’s motivation to contribute to the collective good of the river system. Meanwhile, various upstream and downstream actors manifest individual disparities as a result of the direct reciprocity and asymmetric payoff mechanisms. More specifically, the downstream actors are less willing to cooperate unless there is a high probability that long-term interactions are ensured; however, a greater level of asymmetries is likely to increase upstream actors’ incentives to cooperate even though the interactions could quickly end. The upstream actors also display weak sensitivity to an increase in the total number of actors, which generally results in a reduction in the other actors’ motivation for cooperation. It is also shown that the indirect reciprocity mechanism relaxes the overall conditions for cooperative Nash Equilibrium.

## Introduction

The emergence and evolution of collective cooperation in common-pool resource (CPR) dilemmas have fascinated scholars from various disciplines [1]–[8]. The fundamental puzzle lies in the mechanisms that facilitate costly cooperative and altruistic behavior of individuals who interact with other competitors in rigorous environments. Previous literature on social theory and public good problem has shown that some social mechanisms such as direct reciprocity (repeated interactions) and indirect reciprocity (reputation) can augment the level of collective cooperation [8]–[15]. Yet as much as the underlying structure of CPR dilemmas might be analogous to general public good problems, it is worth noting that a CPR system is unique in the sense of involving both physical and social attributes. These two mutually interconnected dimensions constitute the context in which human beings interact with nature as well as with each other. Therefore, to better understand the dynamics of collective action in governing CPRs, we argue that it is important to simultaneously examine the social and physical characteristics of CPR systems.

One of the main features of CPR systems is individuals’ heterogeneities that are attributed to their physical geographies. For instance, different upstream and downstream actors are heterogeneous in terms of their influences to a river system. They are also diverse in the sense of being victims or beneficiaries who are dependent on other actors’ behavior. However, most general theoretic studies of collective action in CPR dilemmas have been established on an assumption that all actors share symmetric access and position with regard to the commons [2], [4], [16]–[19]. It is worth noting that individual asymmetries might be able to substantially change the structures and results of previous theoretic models. Therefore, it warrants further study on the elements that constitute the asymmetries as well as the internal and external asymmetric mechanisms under which certain regularities might hold.

In this paper, we focus on surface water, which is a controversial CPR that flows across physical boundaries. With a lack of theoretical studies on the asymmetric gains and losses associated with the geographical locations and actions of different upstream and downstream actors, we aim to establish a formal model to investigate how a physical mechanism (asymmetric payoff) and a social mechanism (reciprocity) jointly affect collective cooperation in water sharing interactions. Built upon Raub and Weesie’s [20] model regarding reputation and efficiency in social interactions, our study adds to previous literature in four main aspects. Firstly, we apply a game theoretic model to analyze a specific linear configuration which resembles a river system in the real world. Secondly, we incorporate multi-players into the game theoretic model to demonstrate a more complex group interaction of sharing limited water resources. Thirdly, asymmetric payoffs are integrated with the model to reflect heterogeneous features that are attached to different upstream and downstream actors. Finally, the study simultaneously analyzes the effects of two mechanisms which involve both physical and social attributes of a river system.

This paper first presents an iterative N-person Prisoner’s Dilemma Game (PDG) which enables the direct reciprocity mechanism under which peer punishment could be enforced in future encounters between any two actors. Then with a simple evolutionary approach, we introduce the indirect reciprocity mechanism under which every actor in the game could respond to other actors’ behavior based on the information one has received through a linear system. Also, we incorporate the asymmetric payoff mechanism into the model by modifying the standard PDG payoff matrix. Moreover, we quantitatively address conditions for cooperative Nash Equilibrium (NE) under three different information scenarios. Lastly, we perform numerical simulations in Matlab to illustrate the effects of each independent variable under the equilibrium conditions and provide intuitive explanations on the implications of the asymmetric payoff and reciprocity mechanisms.

## Methods

**Figure 1:**
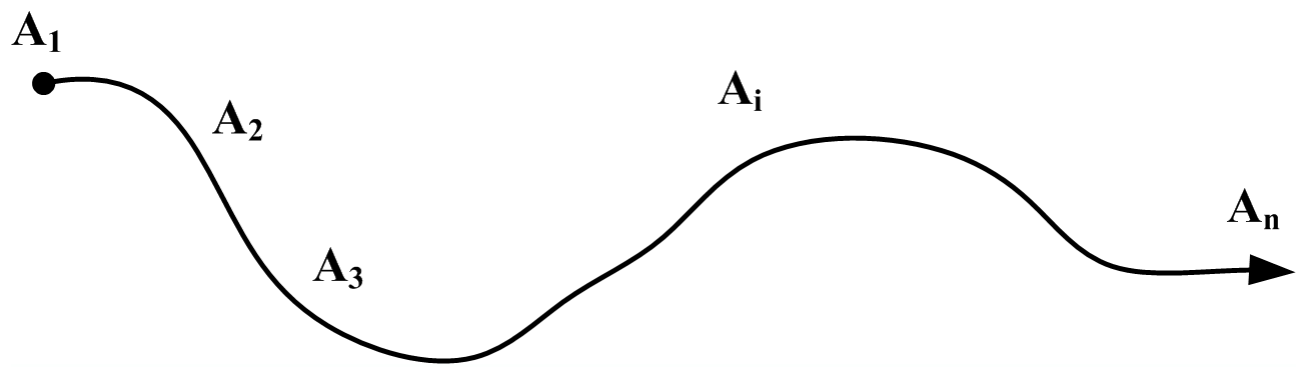
Geographic distribution of water users.

Consider a river system that consists of a finite number of *n* actors (*A_1_, A_2_…A_i_…A_n_*) who are located along the river in a fixed sequence as shown in Figure 1. The subscripts denote geographical locations of the actors. We refer to *A_1_* as the head-end actor and *A_n_* as the tail-end actor. Being confined by the boundaries of the catchment, interactions between all water users are assumed to occur in an abstract linear system. Also, the water users along the river are institutional actors who have to engage in activities of utilizing water resources on a regular basis. Thus they are assumed to be unable to refuse participation; however, there is a probability that the interactions may end, i.e. the decision-makers of the institutional actors might be replaced or purposely move out of the river system. Follow the rational choice theory, the actors are assumed to be selfish who aim at maximizing their payoffs.

We assume that all actors are playing the PDGs on a discrete time scale *(t  =  1, 2, 3…)* till an indefinite end. The event continues with a probability *0<β<1*. Within each event moment *t*, every actor must make a binary choice between contributing to preserve the river environment (C) and inconsiderately exploiting the water resources (D). The choice of every actor is made simultaneously. We assume every actor is fully informed on both alternatives (C or D). We also refer to C as cooperation and D as defection in some of the following analysis. Once the action is made, any actor *A_i_* cannot behave differently towards others within a single event moment. It implies that actor *A_i_* actually plays a large game which is composed of *n-1* pairwise PDGs (supergames) against all other actors within every event moment. By allowing the game to be indefinitely repeated, the direct reciprocity mechanism is thus established in the way that any actor is able to reciprocate with others who had cooperated or defected against himself in potential future interactions.

Then we introduce the indirect reciprocity mechanism by adding an information set to the model. The information set *I_i_* is a profile of other actors’ behavior that any actor *A_i_* obtained. It is a critical element of the indirect reciprocity mechanism; because, in an interconnected social structure, every actor’s present behavior might not only influence his present utility, it also generates a reputation which influences other participants’ actions towards him in their potential future interactions. Therefore, we assume that every actor’s choice of action in each event moment is dependent on the evolving information set that they have obtained during the course of the game.

Next, we introduce the asymmetric payoff mechanism by adding a parameter *α* to the standard PDG payoff matrix as follows,
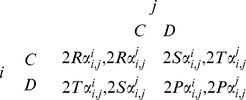


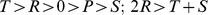



where 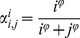, 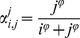, 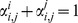; *φ* is a positive real number which indicates the degree of asymmetries; 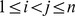, 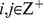. For later reference, we define 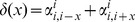 i.e. *j = i−x* or *i+x*, 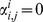 if *j>n* or *j<1*. We also define *γ* = (*T−R*)*/*(*T−P*), where 0<*γ*<1 and *γ* is a constant under the given payoff matrix. Basically, *γ* is an indicator of actors’ short-term incentive for defection.

This modification to the payoff matrix corresponds to the basic physical feature of the river system. That is, any actor’s cooperative behavior will produce a public good, i.e. ecological service to the river system. The public good is shared by all actors, among whom the relative downstream actors benefit more than the upstream ones. Similarly, any actor’s defective behavior will generate a loss to the public good, i.e. environmental degradation. This negative outcome is also shared by all actors, among whom the relative downstream actors lose more than the upstream ones. One might have a mistaken belief that the downstream actors’ behavior cannot affect the upstream actors. As a matter of fact, a river system is a complete ecological unit in which one actor’s behavior affects all other actors one way or another. For instance, the water quality at the river mouth influences fishes that migrate back upstream to mate and reproduce. The sediment deposition and wetlands at the very downstream of the river are also of great ecological value to upstream areas. Therefore, we argue it is safe to assume, as we presented in the payoff matrix, that the relative downstream actors are more vulnerable to the defection made by the upstream actors than the other way around.

It is worth noting that every pairwise interaction is essentially a PDG although the shares that are obtained by every actor are asymmetric. Here 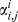 and 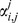 denote the share of utilities that are allocated to actor *A_i_* and actor *A_j_* respectively in their interaction. The asymmetric parameter *α* is adjusted by relative geographical distribution of any two actors *A_i_* and *A_j_*, as well as the exploitation variable *φ*. Here we assume *i<j* and thus actor *A_j_* is located downstream actor *A_i_*. The exploitation variable *φ* represents the degree to which one actor is differentiated from another in terms of utilities allocation. The larger *φ* is the greater asymmetric payoff effect is produced on every actor in the game. In consistent with the ecological characteristics, notice that the tail-end actor is the one that most exposed to the behavior of others, whereas the potential ramifications of other actors’ behavior gradually decreases the further upstream the actors are located.

With the asymmetric payoff mechanism, the long-term expected utilities of any actor is not only dependent on the strategies of their own and the strategies of other actors, it is also dependent on their geographical locations and the asymmetric parameter *α*. Accordingly, we can address the utility function for any actor *A_i_* at a particular moment *t* as follows: 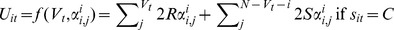
(1)

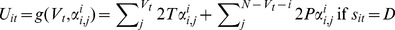
(2)


where *U_it_* denotes the utility of *A_i_* at moment *t*, *s_it_* denotes the action of *A_i_* at moment *t. j∈V_t_*, *V_t_*  =  the numbers which denote the locations of actors who choose C apart from the focal actor *A_i_* at moment *t*. ***N***  =  the numbers which denote the locations of all actors in the game. ***N*** *= (1, 2,…,n),* 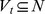. Thus the total utility 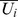 that any actor *A_i_* could receive during the entire game is:
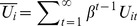
(3)


where 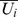 is the exponentially discounted utility sum of *A_i_* from *t = 1* till the indefinite end of the game.

As the game is repeated, we will analyze the game from a simple evolutionary perspective. A desired outcome, from the view of social efficiency, is an equilibrium in which collective cooperation is established. In the scenario of water sharing interactions, actors cannot be ‘dead’ in a biological sense, instead, we assign supergame strategies which allow every actor to update their behavior based on the information they obtain during the course of the game. It is well known that pure strategy C is strictly dominated by D in the PDG. It is also known that unconditional cooperative supergame strategy, which means an actor always chooses C in a supergame regardless of other actors’ choices, is not individually rational because it is strictly dominated by unconditional defective supergame strategy [21], [22]. However, conditional cooperation could be a rational strategy if the choice of C by all actors in all their interactions is the best strategy to use against each other. In other words, collective cooperation might be established when all actors reach an NE in which C is chosen by every actor throughout the game at each moment and no actor can be strictly better off by switching one or more of their supergame strategies given the remaining actors stick with their supergame strategies [20]. There exist countless conditional cooperative supergame strategies as the game is indefinitely repeated. For analytical simplicity, we adopt a classical conditional strategy called “trigger”, which suggests an actor to enter the game with C and then always play D once notified with any defection. The “trigger” is a non-forgiving strategy. One might consider using other conditional strategies that are less strict. Surely more sophisticated defined supergame strategies will enrich the study. However, they will meanwhile extensively increase the complexity of the model. Most importantly, the purpose of this paper is to focus on the effects of asymmetric payoff and reciprocity mechanisms and our following analysis remains valid if other strategies are used. Hence the application of alternative supergame strategies can be discussed in future studies.

## Results

The model is analyzed in three scenarios in which information diffuses through the linear system at different rates. This section aims at addressing conditions for NE in which collective cooperation might be respectively achieved under the three information scenarios. The asymmetric payoff and reciprocity mechanisms are analyzed simultaneously.

### Atomized interactions

We begin with the simplest scenario. Assuming that the information any actor *A_i_* can possibly receive is only from actors who are located adjacent to him (*A_i+1_* and *A_i−1_*). This information is assumed to be received right after an action is committed by *A_i+1_* and *A_i−1_* at moment *t*. Under this specific assumption, interactions are atomized in the sense that an actor only gets information from nobody else but his contiguous actors.

#### Assumption 1

Each actor *A_i_* receives information on the history of their contiguous actors *A_i+1_* and *A_i−1_*, that is for all *i* and *t,*

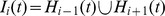



where *I_i_(t)* denotes actor *A_i_*’s information set at moment *t*, *H_i_(t)* denotes the history of *A_i_’*s actions at moment *t*, *H_i_(t) = φ* if *i≤0* or *i>n* or *t≤1*.

Based on non-cooperative game theory, it is already known that either ALL-C (always play C or any other strategy that never initiates a D) or ALL-D (always play D) is a best-response strategy should all other actors use “trigger” [20], [23]. Therefore, to address the necessary and sufficient conditions under which collective cooperation is in NE, one needs to compare any actor *A_i_*’s expected utilities of using ALL-C with his expected utilities of using ALL-D. The conditions for cooperative NE is equivalent to the conditions under which the following inequality stands for any *i = 1,2,…,n*.
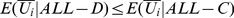
(4)


With the asymmetric payoff matrix, obviously each actor receives different utilities in each event. Yet the expected utilities for any actor *A_i_* of using ALL-C against all other actors who stick with “trigger” is only dependent on their spatial locations regardless of their information situations; because, C will be chosen by every actor throughout the game as no actor will ever initiate a defection according to their supergame strategy (ALL-C or trigger). According to Equation (1), any actor *A_i_*’s expected utilities of using ALL-C is,
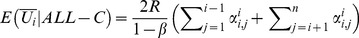
(5)


It is, however, complex to calculate actor *A_i_*’s expected utilities of using ALL-D. The results vary significantly under different information assumptions. Due to the asymmetric payoff mechanism, the results are also greatly dependent on the actors’ geographical locations. We provide a brief summary on the calculation and then derive the conditions for cooperative NE in each information scenario.

Assumption 1 illustrates a situation in which actors are poorly connected by information. For example, if actor *A_i_* initiates a defection in the game at moment *1*, then only actors *A_i−1_* and *A_i+1_* will be aware of the defection and start to defect at moment *2*, the rest of the actors will still choose C. Likewise, only actors *A_i−2_* and *A_i+2_* will realize the defection of *A_i−1_* and *A_i+1_*, and then start to defect at moment *3*, and so forth. In general, defection can only diffuse through contiguity in the atomized system towards both upstream and downstream directions. As actor *A_i_’*s geographical location affects the evolution of the game, the calculation is carried out in two scenarios in which *A_i_* is located either in relative upstream (*i ≤ |n/2|*) or downstream (*i > |n/2|*) the river.

1. If actor *A_i_* uses ALL-D and *i ≤ |n/2|*, then actor *A_n_* will be the last one who realizes that another actor had defected before. Thus actor *A_n_* will start to defect at moment *n-i+1*, from which all actors will always defect afterwards. We divide the total expected utility of *A_i_* into two parts as shown in Equation (6). The first part calculates actor *A_i_’*s utilities before actor *A_n_* turns to defection; the second part is actor *A_i_’*s utilities when there is no cooperative behavior exists in the game.

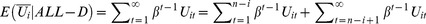
(6)For part one,
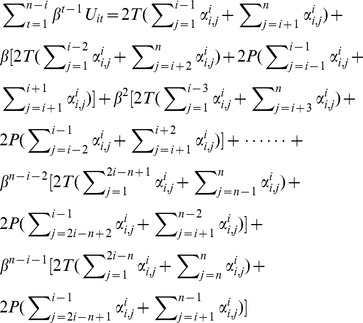



Multiply *β* on each side of the equation and with some basic algebra we will have 
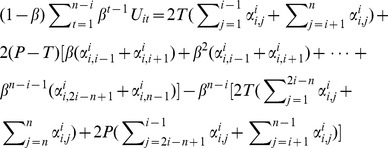
(7)


For part two,
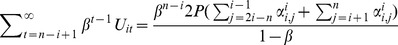
(8)


Combining Equation (7) & (8) and we can solve Equation (6). Put them back to Inequality (4) with Equation (5) we will have,
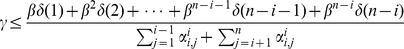
(9)

1. If actor *A_i_* uses ALL-D and *i > |n/2|*, then actor *A_1_* will be the last one who realizes another actor had defected before. Thus actor *A_1_* will start to defect at moment *i*, from which all actors will always defect afterwards. Likewise, we divide the total expected utilities of *A_1_* into the following two parts.

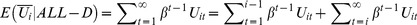
(10)Without repeating a similar calculation as the one presented above, we will have the following equilibrium condition.
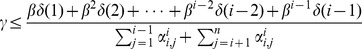
(11)


Summarizing the results of actor *A_i_*’s expected utilities when he is either located upstream or downstream, there is a condition for equilibrium outcome which suggests collective cooperation might be reached under Assumption 1.

#### Condition 1

If every actor uses a trigger against each other in an N-person PDG under Assumption 1, the system is in cooperative Nash Equilibrium if and only if
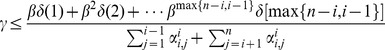



### Perfectly embedded interactions

In this section we relax the first assumption by introducing a system which allows each actor to obtain information from all other actors rather than his neighbors. Besides, we assume the information is perfectly embedded in the system. It implies that every actor receives full information about all other actors’ behaviors immediately after an action is made.

#### Assumption 2

Each actor *A_i_* receives information on the history of all actors in the game, that is for all *i* and *t.*

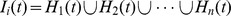



Under assumption 2, the expected utilities of actor *A_i_* if he uses ALL-D are simple to calculate. Because, any defective actor only has one-shot opportunity to abuse other actors’ cooperation and all actors would immediately realize the existence of a defective actor once the defection is made. Then the entire game would turn into full defection. Thus the expected utilities of actor *A_i_* when he chooses ALL-D against trigger are,
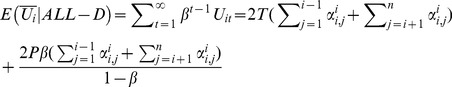
(12)


Put Equations (12) & (5) back to Inequality (4), we have the equilibrium conditions for collective cooperation under Assumption 2.

#### Condition 2

If every actor uses a trigger strategy against each other in an N-person PDG under Assumption 2, the system is in cooperative Nash Equilibrium if and only if 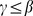



### Imperfectly embedded interactions

Either atomized or perfectly embedded interactions represent a relatively extreme situation of information exchange. In the following section we introduce a more realistic assumption under which information is partly or imperfectly informed to all actors. In particular, for any actor *A_i_*, it is still assumed that information can be immediately received from his contiguous actors. Meanwhile, he can also obtain information about the behavior of any other actor *A_j_* (*i ≠ j*), but only after a certain time lag *π_ij_*, which increases with the distance between actors *A_i_* and *A_j_*.

#### Assumption 3

Each actor *A_i_* immediately receives information on the history of his contiguous actors *A_i+1_* and *A_i−1_*, and is informed with a time delay *π_ij_*>0 on the history of all other actors, that is for all *i* and *t.*

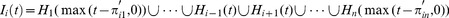



where *π^’^_ij_* is the smallest integer that is strictly larger than *π_ij_*.

Finding the conditions for cooperative NE under Assumption 3 is more complex due to the new parameter *π_ij_*. This assumption allows everyone to receive information from distant actors. The parameter *π_ij_* determines how soon the information can be received. Clearly, the longer time it takes for information to transfer, the fewer actors could have been notified that defections have been made in earlier rounds. More generally, if a defection was made by actor *A_i_*, at moment *1*, for any other actors *A_j_, i ≠j*

If *π^’^_ij_>|i−j|*, then actor *A_j_* will defect at moment *|i−j|*+1;

If *π^’^_ij_≤|i−j|*, then actor *A_j_* will defect at moment *π^’^_ij_*+1;

It is important to note is that Assumption 1 & 2 could be interpreted as two extreme cases of Assumption 3. That is, when *π_ij_*→∞, the information among distant actors travels so slow that the case is the same as atomized interaction; when *π_ij_*→0, the information travels so fast that everyone will immediately be aware of the history of all other actors as in perfectly embedded interactions.

Although the information might travel at different rates under Assumption 3, to find the condition for cooperative NE, we only need to focus on a scenario in which actor *A_i_* gains the highest expected utilities if he uses ALL-D against all other actors who use trigger strategy. Obviously, no rational actor would defect if his highest expected utilities are smaller than his expected utilities of using ALL-C.

To allow actor *A_i_* the highest expected utilities of using ALL-D under Assumption 3, information should only travel slightly faster than that in atomized interactions; because, it will give actor *A_i_* the highest short-term benefits before the whole game turns into universal defection. More specifically, it implies that if *A_i_* initiates a defection at moment *1*, then actors *A_i−1_* and *A_i+1_*, being *A_i_*’s neighbors, will be aware of the defection and start to defect at moment *2*; besides, actors *A_i−2_* and *A_i+2_* will also receive the information about actor *A_i_* and start to defect at moment *2*. Because, the information is better embedded in this scenario than it is in atomized interactions and actors *A_i−2_* and *A_i+2_* are geographically closer to *A_i_* than other actors. Yet we consider *A_i−2_* and *A_i+2_* are the only two more actors who can receive the defective information so that actor *A_i_* is ensured to gain the maximum benefits by using ALL-D under Assumption 3.

The calculation of actor *A_i_*’s expected utilities is consisted of four different scenarios in terms of the geographical location of *A_i_* as well as the number of actors who are located in the upstream and downstream directions of *A_i_*. We present the deduction for one scenario in which actor *A_i_* is located in relative upstream (*i ≤ |n/2|*) and the total amount of downstream actors is an even number (*n−i = 2m, m∈Z^+^*). In this scenario, actor *A_n_* will be the last one who realizes another actor had defected in earlier rounds. Actor *A_n_* will start to defect at moment *(n−i)/2+1*, from which all actors will defect afterwards. Similar to the calculation under Assumption 1, we divide the total expected utilities of actor *A_i_* into two parts as follows.
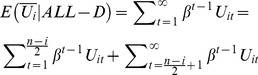
(13)


For part one,
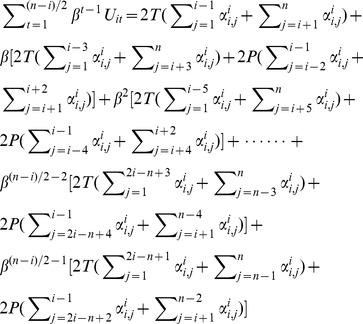



Multiply *β* on each side of the equation and with some basic algebra we will have 
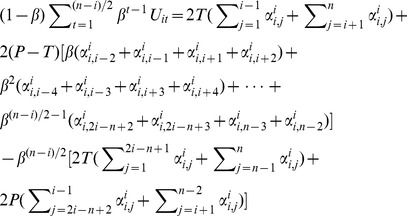
(14)


For part two,
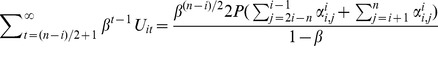
(15)


Combining Equation (14) & (15) and we will solve Equation (13). Put them back to Inequality (4) with Equation (5) we will have,
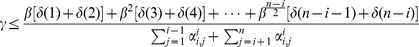
(16)


The other three scenarios are *i ≤ |n/2|*, *n−i = 2m−1, m∈Z^+^*; *i > |n/2| , i = 2m−1, m∈Z^+^* ; and *i > |n/2|,* i = 2m, *m∈Z^+^.* The deductions about these three scenarios are fairly the same as the one we presented above. There are only subtle differences in their results. Without repeating the same procedure, we have the equilibrium conditions for collective cooperation under Assumption 3.

#### Condition 3

If every actor uses a trigger strategy against each other in an N-person PDG under Assumption 3, the system is in cooperative Nash Equilibrium if and only if

when max{n−i, i} = 2m, m∈Z^+^,
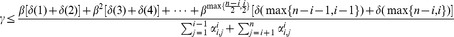



when max{n−i, i} = 2m−1, m∈Z^+^,
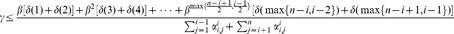



## Discussion

The conditions for cooperative NE under the three assumptions are very complex. It is thus difficult to get intuitive insights into the implications of the equilibrium conditions. However, we are able to find regularities from their mathematical expressions. In particular, all of the three conditions are inequalities which comprise of the “temptation to defect” *γ* on the left and a function on the right, which the latter is dependent on four variables *n, β, φ* and *i*. For analytical simplicity, we respectively define the right side function in condition 1, 2 and 3 as *f_a_*, *f_p_* and *f_im_*. Therefore the conditions for cooperative NE could be translated into the following mathematical expressions, 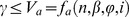
(17)

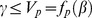
(18)

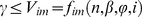
(19)


where *V_a_*, *V_p_* and *V_im_* denote the values of *f_a_*, *f_p_* and *f_im_*.

The three conditions basically imply that cooperative outcomes might be supported under each information assumption, providing that *V_a_*, *V_p_* and *V_im_* are sufficiently large to outweigh the incentive for short-term defection *γ*. Apparently, the larger *V_a_, V_p_* and *V_im_* is, the more likely cooperation is achieved. We should note that the condition for cooperative NE under perfect information is straightforward in the sense of being only dependent on *β* regardless of asymmetries in the system and the geographical location of an actor. Whereas in the other two scenarios, it is difficult to establish explicit understandings of the effects of *n, β, φ* and *i*; because, all of them affect *V_a_* and *V_im_* concurrently. Therefore, we carry out numerical simulations in Matlab to examine how *V_a_* and *V_im_* respond to changes of *n, β, φ* and *i* on a comparative basis. The simulations produce 142,560 data sets under circumstances when *n* varies from 3 to 50 at a 1 interval, *β* varies from 0.01 to 0.99 at a 0.01 interval, *φ* varies from 0.1 to 3 at a 0.1 interval and *i* varies from 1 to *n* at a 1 interval.

To better examine the effects of each independent variable in *f_a_* and *f_im_*, our analysis is designed to hold variables other than the focal variable constant in selective scenarios. We start with the total number of actor *n*. Then the variables *β, φ* and *i* are analyzed under the condition that the total number of actors is fixed to 20.

**Figure 2:**
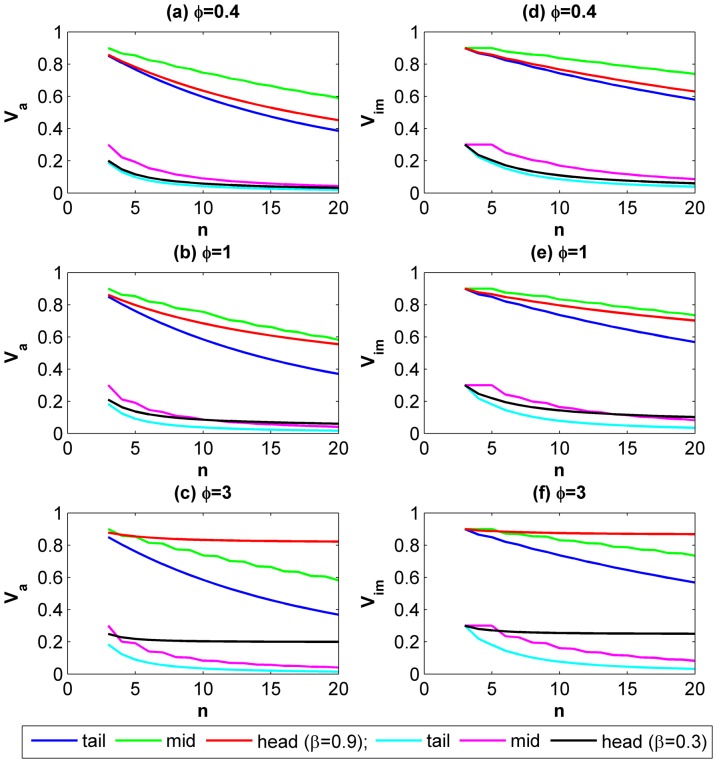
The effect of total number of actors *n* on the conditions for cooperative NE. (a, b, c) *V_a_* for the head-end, mid-stream and tail-end actors in atomized interactions when *φ = 0.4, 1* and *3* respectively; (d, e, f) *V_im_* for the head-end, mid-stream and tail-end actors in imperfectly embedded interactions when *φ = 0.4, 1* and *3* respectively. The curves provide each actor’s general response to the increase of the group size. Each actor’s motivation for cooperation is represented by two curves *β = 0.3* and *0.9* in every subplot.

Figure 2 indicates how *V_a_* and *V_im_* would change as the total number of actors *n* increases. We considered three scenarios in which the level of asymmetry *φ* is set to 0.4, 1 and 3.We select the head-end, tail-end and midstream actors as examples to illustrate. In each subplot, we also compare *V_a_* and *V_im_* under two different continuing probability 0.3 and 0.9. In general, the results demonstrate a descending trend of *V_a_* and *V_im_* in most scenarios. The results correspond to Olson’s influential argument about “the logic of collective action” which states that the larger a group is, the less likely they are to create social incentives which lead its members to provide collective goods [11]. Nonetheless, we also discover that the head-end actor’s incentive to cooperate is hardly affected by the group size when a high level of asymmetries exists during the course of interactions. An intuitive explanation for this phenomenon is that the head-end actor has most control over his potential loss. Greater level of asymmetries reduces his dependence on other actors’ behavior. Hence his risk of being defected by others does not increase with the number of actors involved.

**Figure 3:**
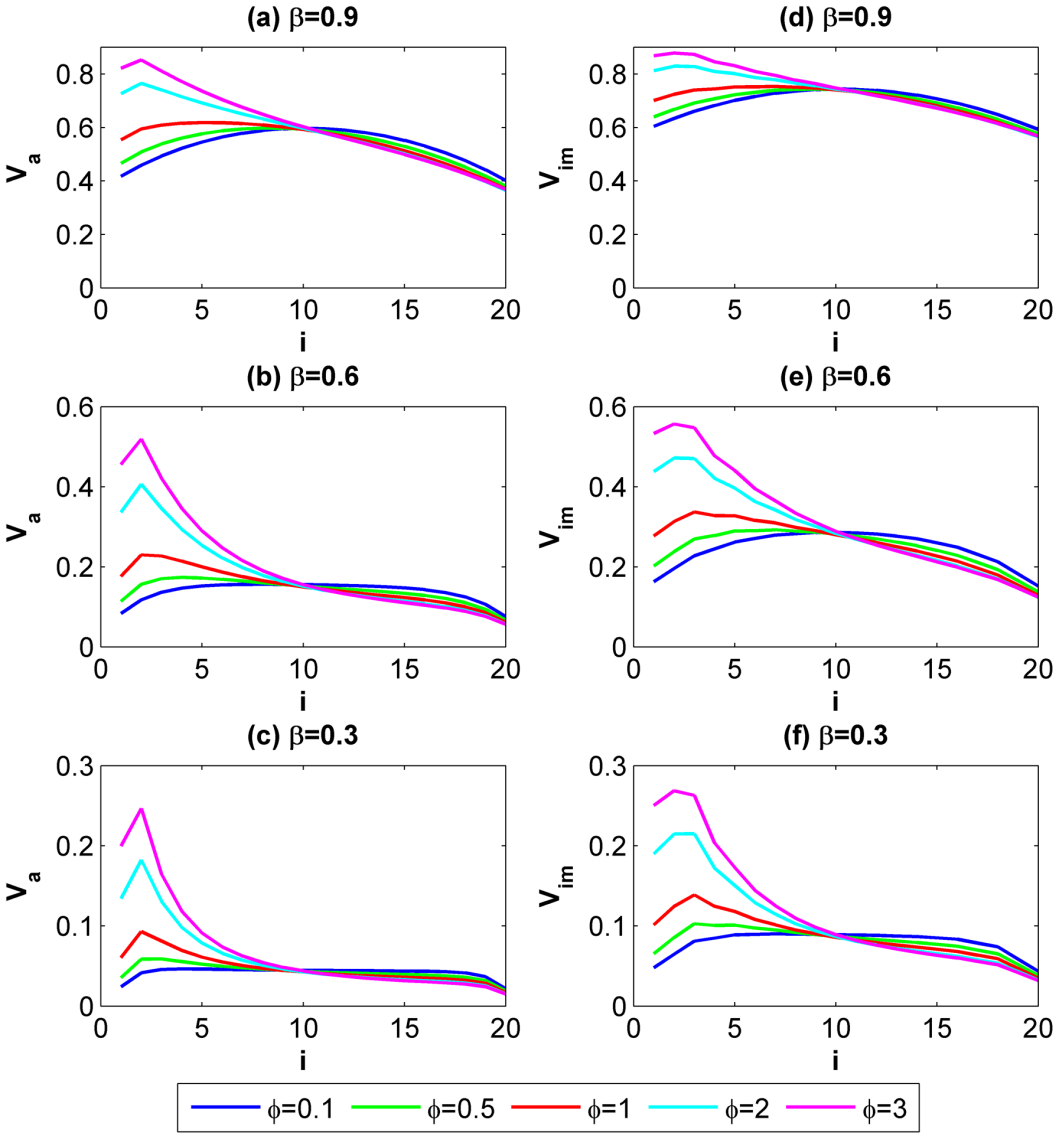
The effect of actors’ geographical locations *i* on the conditions for cooperative NE when the group size is fixed to 20. (a, b, c) *V_a_* for all of the 20 involved actors in atomized interactions when *β = 0.9, 0.6* and *0.3* respectively; (d, e, f) *V_im_* for all of the 20 involved actors in imperfectly embedded interactions when *β = 0.9, 0.6* and *0.3* respectively. The curves provide a comparison in terms of motivation for cooperation for all actors who are located at different positions on the river. Each actor’s motivation for cooperation is also compared in every subplot when the interactions take place under different levels of asymmetries *φ = *0.1, 0.5, 1, 2 and 3.

Figure 3 indicates how *V_a_* and *V_im_* would change as an actor’s geographical location *i* moves gradually from the source to the end of the river. We select three scenarios in which the continuing probability *β* is set to 0.3, 0.6 and 0.9. In each subplot, we also compare how each actor reacts to variations of the levels of asymmetric payoff under the circumstances when *φ* equals to 0.1, 0.5, 1, 2 and 3. Two remarks can be drawn from this figure. First, *V_a_* and *V_im_* increase with *i* at the beginning and then decrease after *V_a_* and *V_im_* reach their apexes. Although the position of the apexes varies with *φ* and *β*, it implies that relative up-midstream actors are more likely to cooperate than the others when the remaining variables are held invariant. Second, the upstream curves in each subplot become steeper as *φ* increases. It shows that greater individual differences exist among upstream actors with higher levels of asymmetries.

**Figure 4:**
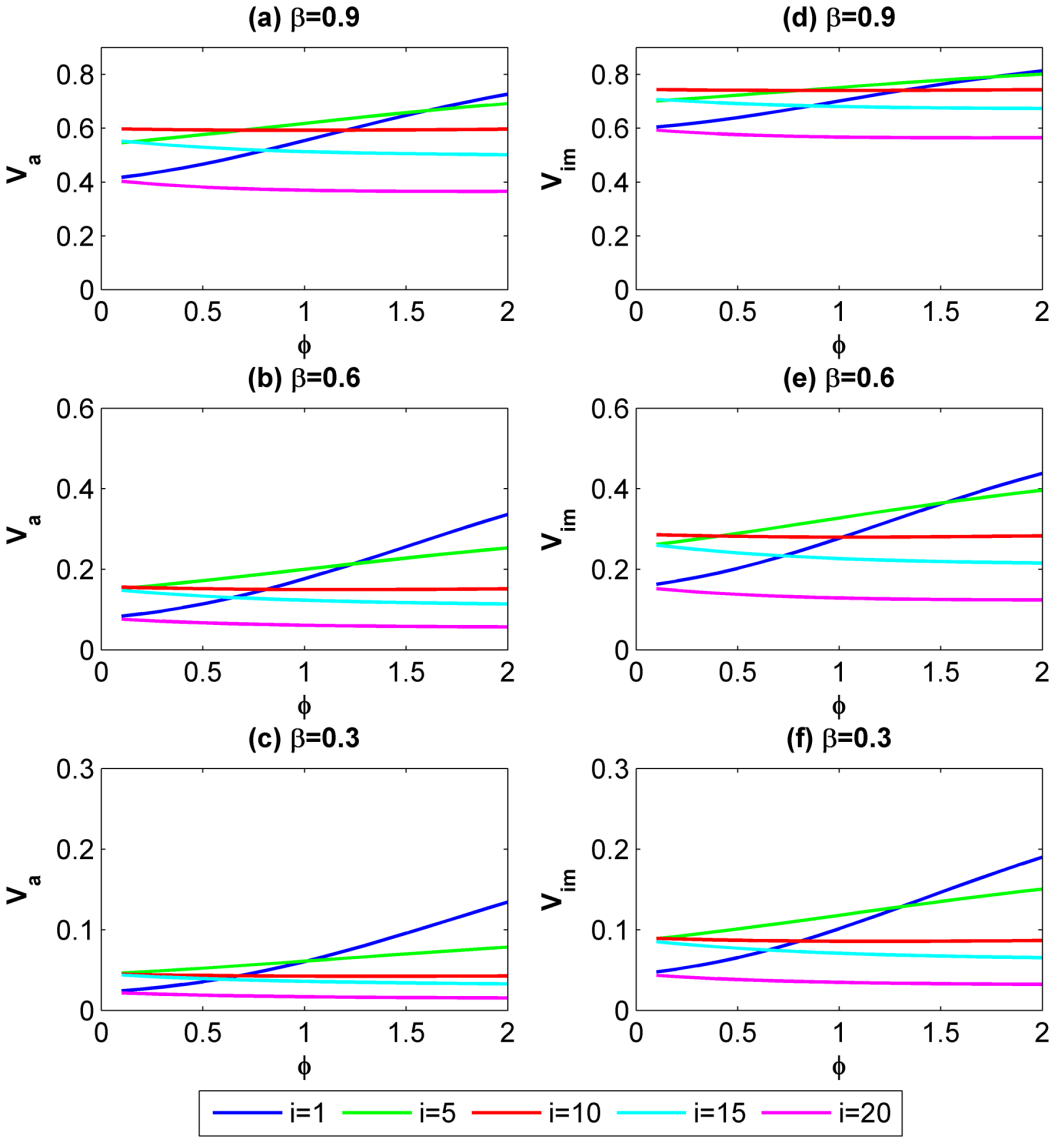
The effect of the level of asymmetries *φ* on the conditions for cooperative NE when the group size is fixed to 20. (a, b, c) *V_a_* for five involved actors (head-end, mid-upstream, mid, mid-downstream, tail-end) in atomized interactions when *β* = 0.3, 0.6 and 0.9 respectively; (d, e, f) *V_im_* for five involved actors (head-end, mid-upstream, mid, mid-downstream, tail-end) in imperfectly embedded interactions when *β* = 0.3, 0.6 and 0.9 respectively. The curves provide each actor’s general response to the increase of the level of asymmetries.

Figure 4 indicates how *V_a_* and *V_im_* would change as the level of asymmetries *φ* increases. Likewise, we select three scenarios in which the continuing probability *β* is set to 0.3, 0.6 and 0.9. We compare the motivation for cooperation of five different actors (head-end, mid-upstream, mid, mid-downstream, tail-end). It is shown from Figure 4 that *V_a_* and *V_im_* for the head-end and mid-upstream actors increase with *φ*, yet for downstream actors they decrease slightly with *φ* and tend to stabilize though *φ* continues to increase. It implies upstream actors are sensitive to the degree of asymmetries and more likely to cooperate than downstream actors when higher levels of asymmetries display. An intuitive explanation for the joint effect of *φ* and *i* is the actors’ reactions to risks. As the level of asymmetries increases, the upstream actors have less reservation about the risk of being hurt by others and thus tend to cooperate; whereas the situation is reversed for the downstream actors who bear increasing risks thus their motivation for cooperation tend to maintain at a low level.

**Figure 5:**
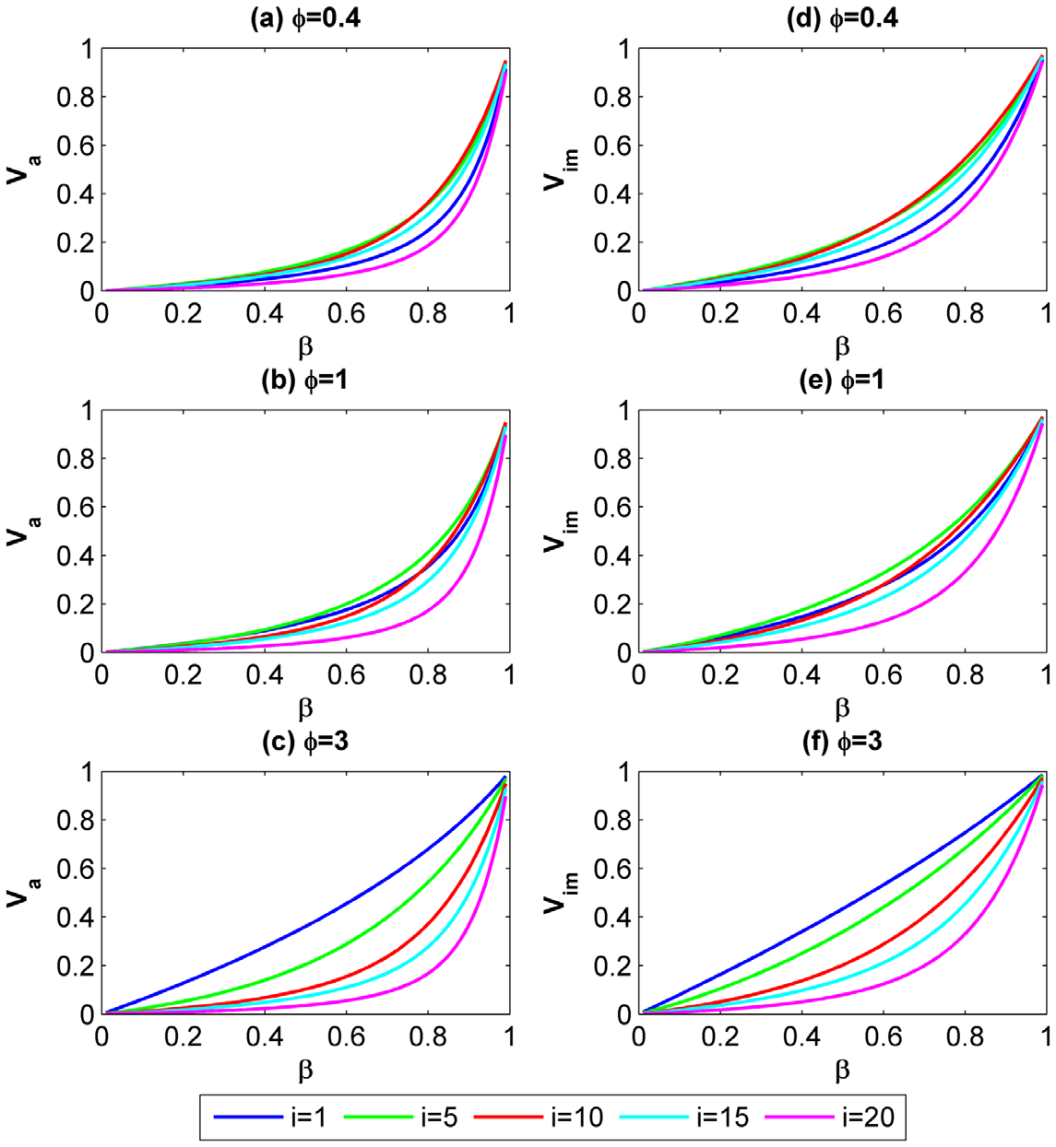
The effect of continuing probability *β* on the conditions for cooperative NE when the group size is fixed to 20. (a, b, c) *V_a_* for five involved actors (head-end, mid-upstream, mid, mid-downstream, tail-end) in atomized interactions when *φ* = 0.4, 1 and 3 respectively; (d, e, f) *V_im_* for five involved actors (head-end, mid-upstream, mid, mid-downstream, tail-end) in imperfectly embedded interactions when *φ* = 0.4, 1 and 3 respectively. The curves provide each actor’s general response to the increase of the continuing probability *β*.

Figure 5 indicates how *V_a_* and *V_im_* would change as the continuing probability *β* increases. We consider three scenarios in which the level of asymmetries *φ* is set to 0.4, 1 and 3. In each subplot, we compare the motivation for cooperation of five different actors (head-end, mid-upstream, mid, mid-downstream, tail-end). The value of *β* is generally referred as “the shadow of the future” which indicates the possibility of future interactions between all involved actors. Hence, the larger *β* is the more likely the game can continue. We can draw two remarks from Figure 5. On one hand, for a particular actor, *V_a_* and *V_im_* increase with *β* when is *φ* constant. It confirms that an actor is more likely to cooperate when “the shadow of the future” is more significant. On the other hand, the slope of the curves for downstream actors becomes steeper than upstream actors when *β* is relatively large; the situation is reversed when *β* is relatively small. It implies that downstream actors’ motivation for cooperation increase faster than upstream actors when there is a higher possibility that future interactions will continue taking place. To the contrary, upstream actors are more motivated to cooperate than downstream actors even when there is a greater chance that the game could quickly end. An intuitive explanation for the joint effects of *β* and *i* is actors’ vision for their long-term interactions. For the downstream actors, assurance of future interactions will reduce their risks by giving them more control over other actors. They will therefore more likely to provide cooperation. This effect is amplified by the level of asymmetries. Whereas upstream actors are less exposed to others hence direct reciprocate behavior would not reduce their motivation for cooperation.

**Figure 6:**
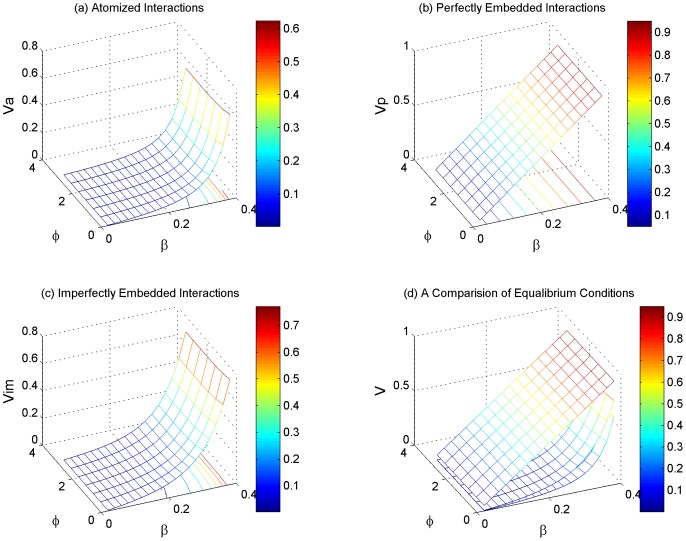
Overall conditions for cooperative NE when the group size is fixed to 20. (a) conditions for cooperative NE in atomized interactions; (b) conditions for cooperative NE in perfectly embedded interactions; (c) conditions for cooperative NE in imperfectly embedded interactions; (d) comparison of the conditions for cooperative NE under Assumption 1, 2 & 3.

After analyzing the effect of each variable individually, in Figure 6 we present the overall conditions for cooperative NE under Assumption 1, 2 & 3 in three dimensional graphs. Instead of providing a condition for cooperation that is applicable to any actor *A_i_* in the game, we located the minimum *f_a_* and *f_im_* with respect to *i* by screening our massive amount of simulation results. This approach helps us find the single one actor who has the least incentive for cooperation when other variables (*n*, *β* and *φ*) are held invariant. The results confirmed that the tail-end actor is the one that shows least willingness to choose C. In other words, the conditions for cooperative NE should always stand if we apply *i = n* in all the three assumptions above. In that case, *f_a_* and *f_im_* become functions of *n*, *β* and *φ*. Consider the group size *n = 20*, we generate the overall cooperative NE conditions as shown in Figure 6. The comparison of cooperative NE conditions reveals the effect of indirect reciprocity mechanism. Specifically, the cooperative NE condition in perfectly embedded interactions is only dependent on *β* and thus is a flat surface in Figure 6(b) regardless of other variables. The flat surface is always above the other two curved surfaces in Figure 6 (a) & (c), among which the former represents atomized interactions and the latter represents imperfectly embedded interactions. Figure 6(d) illustrates that the effect of indirect reciprocity mechanism and demonstrates that the conditions for collective cooperation become less restrict when information is better embedded in the game.

## Conclusions

In this paper we simultaneously investigate the effects of asymmetric payoff and reciprocity mechanisms on collective cooperation in water sharing interactions. We establish a quantitative model of iterative N-person PDG and study the game as it evolves with all actors’ actions which are conditional on their available information during the course of the game. Under different information scenarios, our analysis produces conditions for NE in which collective cooperation is likely to be established. The results suggest that the direct reciprocity, or put it poetically “the shadow of the future”, can increase all actors’ motivation to contribute to the collective good. Meanwhile, various upstream and downstream actors manifest individual disparities as a result of the direct reciprocity and asymmetric payoff mechanisms. More specifically, the downstream actors are less willing to contribute unless there is a high probability that long-term interactions are guaranteed; however, a greater level of asymmetries is more likely to increase upstream actors’ incentives to cooperate even though the interactions could quickly end. The upstream actors also display weak sensitivity to an increase in the total number of actors, which generally results in a reduction in the other actors’ motivation for cooperation. It is also shown that the indirect reciprocity mechanism relaxes the overall conditions for cooperative NE.

In general, our model is a preliminary theoretical attempt to connect the asymmetric payoff with reciprocity mechanisms. We endeavor to examine their joint effects on the collective behavior of heterogeneous selfish actors in a theoretical river system. We generate theoretical predictions based on the N-person iterative asymmetric PDG. In this paper, we do not intend to conclude with a deterministic argument about a causal relationship between the two mechanisms and collective cooperation in water governance. Obviously, a lot more theoretical models and empirical case studies remain to be conducted. However, we do expect to provide a more comprehensive perspective on the theory of collective cooperation, which emphasizes the integrity of CPR systems in the sense of including both physical and social characteristics.
